# Clear Aligner Therapy and Marginal Edge Design: Clinical and Laboratory Evidence on Periodontal and Biological Outcomes—A Scoping Review

**DOI:** 10.3390/dj14030130

**Published:** 2026-02-24

**Authors:** Emilia (Prodea) Rusu, Ana-Petra Lazăr, Bianca Luminița Erhan, Eugen Bud, Mariana Păcurar, Luminița Lazăr

**Affiliations:** 1 Doctoral School (IOSUD), George Emil Palade University of Medicine, Pharmacy, Science, and Technology of Targu Mures, 38 Gheorghe Marinescu Street, 540139 Targu Mures, Romania; rusu.emilia.24@stud.umfst.ro; 2 Department of Oral Rehabilitation and Occlusology, George Emil Palade University of Medicine, Pharmacy, Science, and Technology of Targu Mures, 38 Gheorghe Marinescu Street, 540139 Targu Mures, Romania; 3 Doctoral School, Faculty of Medicine, Lucian Blaga University of Sibiu, 2A Lucian Blaga Street, 550169 Sibiu, Romania; 4 Department of Orthodontics, George Emil Palade University of Medicine, Pharmacy, Science, and Technology of Targu Mures, 38 Gheorghe Marinescu Street, 540139 Targu Mures, Romania; eugen.bud@umfst.ro (E.B.); mariana.pacurar@umfst.ro (M.P.); 5 Department of Periodontology, George Emil Palade University of Medicine, Pharmacy, Science, and Technology of Targu Mures, 38 Gheorghe Marinescu Street, 540139 Targu Mures, Romania; luminita.lazar@umfst.ro

**Keywords:** biofilm, dental plaque, gingivitis, microbiota, orthodontic appliances, removable, periodontal diseases

## Abstract

**Background:** Clear aligner (CA) therapy has been increasingly adopted as an alternative to fixed orthodontic appliances. However, evidence regarding its periodontal effects, including gingival inflammation, biofilm composition, and the potential role of aligner marginal edge design, remains insufficiently mapped. The objective of this scoping review was to map and synthesize available clinical and laboratory evidence on periodontal and biological outcomes associated with CA therapy, with particular attention to the potential influence of aligner marginal edge design. **Methods:** This scoping review followed PRISMA-ScR and the Population–Concept–Context (PCC) framework. PubMed, Scopus, and Web of Science were searched from 1 January 2015 to 24 October 2025. A total of 1587 records were identified; after deduplication, 770 unique records were screened by title and abstract, followed by full-text assessment of potentially eligible articles. Twenty-five primary studies published between 2020 and 2025 met eligibility criteria and were included in the review. **Results:** The final synthesis comprised clinical investigations, laboratory studies, and case reports. Clinical periodontal indices and inflammatory biomarkers were assessed using heterogeneous protocols and timepoints. Only three studies specifically addressed aligner marginal edge design; one directly compared margin configurations, reporting differences in local gingival response, with substantial diversity in study design precluding quantitative synthesis. **Conclusions:** Available evidence on periodontal outcomes during CA therapy is methodologically heterogeneous. Investigations of aligner marginal edge design remain scarce. Standardized assessment protocols and targeted clinical studies are needed to establish the periodontal relevance of trimline configurations.

## 1. Introduction

In the past decade, orthodontic treatment using clear aligners (CAs) has become an increasingly common alternative to conventional fixed appliances, with growing adoption reflecting increased patient demand for more esthetic and comfortable treatment options that facilitate oral hygiene maintenance [1,2].

Although the esthetic and functional advantages of aligners are well documented, their impact on periodontal health remains an area of active investigation [3]. Unlike fixed appliances, aligners are removable and may permit more effective plaque control [4]. However, their trimline configuration (scalloped, straight-cut, or hybrid) may influence local gingival response and biofilm dynamics [5]. Clinical findings remain mixed: some studies report improvements in periodontal parameters [6,7,8], while others document variations in inflammatory or microbiological markers [9,10,11]. Laboratory investigations have further examined bacterial adherence to aligner materials and surface modifications aimed at reducing microbial colonization [12,13].

The aim of this scoping review is to map and synthesize clinical and laboratory evidence on the periodontal and biological effects of CA therapy, with particular attention to aligner marginal edge design.

Specifically, this review addresses the following research question: What is the impact of CA therapy on periodontal health, and what evidence exists regarding the influence of aligner marginal edge design on periodontal outcomes?

## 2. Methodology

This synthesis focused on identifying thematic patterns, methodological approaches, and existing knowledge gaps, rather than evaluating comparative effectiveness, causality, or clinical superiority. Data were synthesized descriptively without formal comparison between interventions.

The present review was structured according to the Population–Concept–Context (PCC) framework recommended by the Joanna Briggs Institute for scoping reviews [14,15]. The PCC framework provides a structured approach to defining eligibility criteria in scoping reviews, where Population describes the important characteristics of participants, Concept encompasses the core elements under examination (which may include interventions, phenomena of interest, or outcomes), and Context specifies the setting and relevant circumstances.

The population of interest included adolescent and adult patients, aged 12 years or older, undergoing orthodontic treatment with CA. The central concept addressed aligner therapy and marginal edge design (scalloped, straight-cut, or hybrid) in relation to periodontal parameters, inflammatory biomarkers, and microbiological characteristics. The context considered both clinical and laboratory investigations published between 2020 and 2025 within the interdisciplinary fields of orthodontics and periodontology.

This review was conducted in accordance with the PRISMA-ScR (Preferred Reporting Items for Systematic Reviews and Meta-Analyses extension for Scoping Reviews) guidelines to enhance methodological transparency and consistency with current reporting standards for scoping reviews.

### 2.1. Study Type and Aim

The searches aimed to identify clinical and laboratory studies reporting periodontal parameters (plaque index (PI), gingival index (GI), bleeding on probing (BOP), probing pocket depth (PPD), and clinical attachment level (CAL)), inflammatory markers (IL-1β, TNF-α, MMP-8, and CRP), or microbiological characteristics (biofilm, bacterial load, and adhesion patterns associated with different margin designs).

Study selection was performed in collaboration with a research team through a well-structured process in successive stages: database searches, deduplication, title and abstract screening, full-text assessment, and final inclusion.

### 2.2. Search Strategy

The search was performed across the PubMed, Scopus, and Web of Science databases. The database search covered 1 January 2015 to 24 October 2025 to capture both earlier foundational and recent evidence; however, in line with predefined eligibility criteria, the final included studies were published between 2020 and 2025.

The search strategy combined terms for orthodontic treatment with CA and periodontal or inflammatory indicators and was limited to human clinical studies and laboratory studies using human participants or human-derived biological samples, published in English. Romanian-language records were also screened to minimize language bias; however, none met the final inclusion criteria.

The PubMed (MEDLINE) search combined MeSH terms with free-text keywords, and full texts were retrieved when available. Terms describing orthodontic treatment with aligners included the MeSH descriptor “Orthodontic Appliances, Removable” and keywords such as “clear aligner”, “orthodontic aligner”, and “Invisalign”. These were combined with terms related to periodontal health and gingival inflammation, including “gingival diseases”, “periodontal diseases”, “gingival inflammation”, “PI, GI, BOP, PPD, and CAL”, as well as microbiological terms such as “biofilm, bacterial adhesion, microbiome, bacterial load, cytokines, IL-1β, TNF-α, and MMP-8”.

To include articles investigating the morphological aspects of aligners, the search was expanded to terms referring to margin design—”scalloped, straight cut, trimline, marginal finish, edge design, and gingival margin”—to identify studies addressing the potential influence of margin design on gingival tissues.

Detailed database-specific search strategies and screening steps are provided in the Supplementary Materials [16].

### 2.3. Study Integration and Selection

The search performed in the PubMed (MEDLINE), Scopus, and Web of Science databases generated a total of 1587 records. After deduplication using the Zotero reference manager, 770 unique records remained for screening. The selection of articles was performed in two successive stages.

In the first stage, records were screened by title and abstract for relevance to periodontal outcomes associated with CA therapy. In the second stage, full-text assessment was conducted for potentially eligible articles. Secondary research articles (systematic reviews and meta-analyses) were excluded from the final inclusion set; however, their reference lists were screened when relevant.

As this is a scoping review, the purpose was to map the breadth of available evidence rather than to formally appraise methodological quality or estimate effect sizes. Therefore, no risk-of-bias assessment, critical appraisal, or meta-analysis was performed. A review protocol was not prospectively registered, consistent with Joanna Briggs Institute guidance for scoping reviews focused on evidence mapping rather than comparative effectiveness assessment.

Full-text assessment was based on the following inclusion criteria: clinical studies (randomized, cohort, case–control, or cross-sectional) or laboratory studies using human participants or human-derived biological samples; adolescent or adult participants (≥12 years old); orthodontic treatment with CA; and reporting of one or more periodontal or gingival clinical indicators (PI, GI, BOP, PPD, and CAL) or biological parameters (inflammatory biomarkers, microbiological characteristics). When reported, information regarding aligner marginal edge design (scalloped, straight-cut, or hybrid) was extracted and synthesized.

Study selection was initially conducted by one reviewer following the predefined protocol. To minimize selection bias, the entire screening process, including title/abstract screening and full-text eligibility assessment, was subsequently re-screened by additional members of the research team, strictly adhering to the same methodological criteria. Any discrepancies were discussed and resolved through consensus.

The exclusion criteria targeted articles lacking periodontal data, information on the research methodology, or results; studies focused exclusively on comfort, esthetics, pain, speech, or tooth movement mechanics; editorials, letters, or conference abstracts; and articles from unrelated medical fields (orthopedics, ophthalmology, molecular biology, optics, etc.). Single case reports were generally excluded; however, those providing clinically or biologically relevant information on periodontal outcomes were retained for descriptive mapping purposes.

### 2.4. Data Charting and Synthesis

Data from included studies were systematically extracted using a standardized charting form developed by the research team. For each study, the following information was recorded: first author and publication year; study design and setting; population characteristics (sample size, age range); intervention details (aligner type, marginal edge design when specified, treatment duration); periodontal outcomes measured (clinical indices: PI, GI, BOP, PPD, CAL; and biological parameters: inflammatory markers, microbiological characteristics); key findings; and study conclusions.

The extracted data were synthesized descriptively and organized thematically according to outcome type (clinical periodontal indices, inflammatory biomarkers, microbiological analyses) and study design (clinical trials, observational studies, laboratory investigations). This approach allowed for comprehensive mapping of the evidence landscape while acknowledging the methodological heterogeneity that precluded quantitative synthesis.

## 3. Results

**Figure 1:**
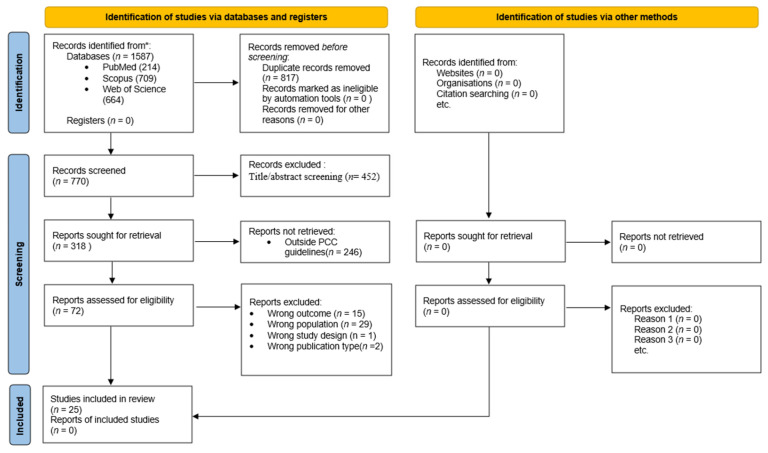
Flow diagram of the search, deduplication, and study selection process. * Databases: PubMed, Scopus, and Web of Science.

A total of 25 eligible articles were identified and analyzed, with the final search conducted on 24 October 2025. Studies were grouped by outcome type: clinical periodontal indices, inflammatory biomarkers, and microbiological analyses. The study selection process is shown in Figure 1.

### 3.1. Study Characteristics and Thematic Grouping

The eligible articles were grouped into three thematic domains: gingival/periodontal inflammation, biofilm and inflammatory markers, and aligner margin design. The distribution of these studies and their corresponding fields of analysis are presented in Table 1. This thematic grouping organized studies across overlapping research areas and highlighted differences in study focus regarding periodontal outcomes. Only a small subset of studies addressed aligner margin design, primarily from a clinical design perspective, with periodontal outcomes reported indirectly or qualitatively.

### 3.2. Study Designs and Methodological Diversity

Beyond thematic classification, the methodological characteristics of the included articles were examined to highlight diversity in study design and approaches used in investigating the association between aligner use and periodontal health (Table 1).

Based on study design and methodological approach, the eligible articles were grouped into four main categories:

(i) Prospective and observational clinical studies;
(ii) Experimental and laboratory studies (in vitro and ex vivo);
(iii) Case reports with high clinical relevance;
(iv) Interdisciplinary studies combining orthodontics, periodontology, and molecular biology.

#### 3.2.1. Prospective and Observational Clinical Studies

Fourteen articles employed a prospective or observational design, including comparisons between CA therapy and fixed orthodontic appliances. These studies monitored parameters such as the evolution of periodontal biomarkers, biofilm dynamics, microbiological and immunological changes, and clinical outcomes at different stages of orthodontic treatment. The main characteristics of these studies are summarized in Table 2.

#### 3.2.2. Experimental and Laboratory Studies (In Vitro and Ex Vivo)

Five experimental studies were identified, focusing on the analysis of cytotoxicity, surface alterations of aligner materials, and the evaluation of molecular markers using laboratory methods (enzyme-linked immunosorbent assay (ELISA), Polymerase Chain Reaction (PCR), and microbial cultures performed on aligner materials or gingival crevicular fluid (GCF)) (Table 3)**.**

These studies reported data on bacterial adhesion, surface characteristics of aligner materials, and inflammatory cytokine expression (IL-1β, TNF-α, MMP-8) under different experimental conditions. Variations in inflammatory marker levels were reported across observation periods.

#### 3.2.3. Case Reports with Clinical and Interdisciplinary Characteristics

Three articles presented complex clinical cases, including severe gingival recessions and interdisciplinary treatment approaches, with periodontal parameters documented in these clinical contexts (Table 4).

#### 3.2.4. Studies with an Interdisciplinary Orthodontics–Periodontology–Molecular Biology Approach

Ten studies noted collaborations between orthodontists and periodontists, as well as specialists in biochemistry or microbiology, integrating clinical evaluations with the analysis of inflammatory biomarkers, microbial sequencing, cytokine determination in GCF, and, in some cases, genomic associations (Table 5).

Across the included studies, periodontal assessment was conducted using a wide range of clinical indices and scoring methodologies. Plaque accumulation was evaluated using different PI, including the Silness–Löe index, the Turesky modification of the Quigley–Hein index, and the O’Leary PI, while GI was primarily assessed using the Löe–Silness GI or modified GI scales. BOP was reported using either percentage-based measures or categorical scoring systems, and PPD and CAL were assessed in selected investigations. Periodontal evaluations were performed at variable timepoints, including baseline, 1, 3, 6, and 12 months. This variability in index selection, scoring systems, and assessment timing limited direct cross-study comparability of reported periodontal outcomes.

One investigation specifically compared aligner margin configurations, with Favero et al. [5] reporting significant differences in periodontal indices between vestibular and juxtagingival rim designs over six months.

CAL was assessed in eight studies focused on adult patients or those with pre-existing periodontal considerations. Seven investigations reported stable CAL throughout treatment, with Leibovich et al. [20] describing favorable outcomes in a case series employing CA for root repositioning prior to periodontal surgery in patients with lower incisor gingival recession. The observed variability in periodontal assessment protocols, scoring systems, and examination timing complicates direct cross-study comparisons.

### 3.3. Influence of Aligner Margin Design

Favero et al. [5] compared vestibular rim (extending approximately 3 mm beyond the gingival margin) versus juxtagingival rim (following the gingival outline) designs in adolescent patients (*n* = 43, aged 14–18 years). Over a six-month observation period, juxtagingival margins were associated with significantly worse periodontal indices (PI *p* = 0.011, GI *p* = 0.03, gingival bleeding index *p* = 0.014), while vestibular rim margins showed no significant changes in any measured parameter.

Rouzi et al. [18] examined oral microbiota and health parameters in aligner patients using 16S rRNA gene sequencing, documenting changes in microbial community composition over three months. The study noted the scalloped design of Invisalign margins in discussion but did not systematically investigate the influence of margin configuration on periodontal outcomes.

Kredig et al. [11] investigated periodontal inflammatory biomarkers in adolescents undergoing aligner therapy with scalloped juxtagingival margins, discussing the potential clinical relevance of trimline configuration based on comparative literature.

Across these studies, the available evidence on aligner margin configuration remains limited and methodologically diverse, with variations in study design, sample populations, assessment protocols, and categorization approaches. These findings should be interpreted descriptively as a mapping of reported associations, without inferring comparative effectiveness or establishing causal relationships.

### 3.4. Temporal and Demographic Distribution

The included articles were published between 2020 and 2025, with publication activity increasing from 2020 onwards. The time frame of the reported investigations was generally close to the publication date (with differences of approximately 1–2 years).

Population characteristics and sample variability were also examined, the number of participants varied among studies, reflecting the diversity in research designs and objectives. In clinical studies, sample size generally ranged from 12 to 90 patients, with most investigations conducted on small cohorts subjected to close longitudinal monitoring. One study included larger samples, reporting up to 146 recessions or multiple cases per patient, depending on the periodontal parameters assessed [7].

From a demographic perspective, the study populations mainly consisted of young adults (approximately 20–35 years) and adolescents (12–18 years). Some studies focused exclusively on adult patients to describe reported periodontal parameters during aligner treatment [30], while others concentrated on adolescent populations to examine periodontal indices and margin design effects [5] or inflammatory biomarkers [11].

## 4. Discussion

In this review, biological outcomes refer to inflammatory, microbiological, molecular, and cellular responses associated with CA, rather than definitive clinical endpoints. These outcomes are discussed in relation to the predefined aim of this scoping review, namely to map how periodontal and biological responses to CA therapy and marginal edge design have been reported across clinical and laboratory studies.

Several clinical studies reported PI, GI, and BOP during CA therapy [18]. In a longitudinal comparison between aligner and fixed appliance treatments, Lombardo et al. [34] reported differences in subgingival microbiota composition across follow-up intervals, with microbial assessments performed at 3 and 6 months in the fixed-appliance group and corresponding periodontal evaluations in the aligner group.

Microbiological studies identified both commensal and pathogenic species on aligner surfaces and reported compositional changes during therapy; these findings are constrained by methodological heterogeneity and the frequent absence of parallel clinical or biomarker assessments [28,34].

Across the included clinical studies, periodontal parameters during aligner therapy were described in relation to fixed appliances using variable study designs, follow-up durations, and oral hygiene protocols, which limited direct cross-study comparability. Some investigations reported variations in specific parameters, such as PI or GI, particularly in interdisciplinary clinical settings and among patients with distinct periodontal phenotypes [17].

The findings of this scoping review should be considered within the broader evidence base comparing periodontal outcomes between CA and fixed appliances. Previous meta-analyses have reported statistically more favorable periodontal indices in patients treated with CA. Jiang et al. [35] identified significantly lower PI and GI in aligner patients, although trial sequential analysis indicated insufficient sample size to support conclusions regarding PPD. Similarly, Llera-Romero et al. [36], in the first meta-regression addressing this topic, reported that periodontal advantages associated with CA increased with treatment duration and described a markedly reduced risk of white spot lesions.

More recent reviews have tempered these findings. Di Spirito et al. [37] concluded that reported differences between aligners and fixed appliances, although statistically significant, were clinically negligible, while Crego-Ruiz and Jorba-García [38] found insufficient evidence to support definitive superiority of CA therapy. Importantly, none of these syntheses evaluated the potential influence of aligner marginal edge design on periodontal outcomes, representing a gap that the present scoping review sought to address. Consistent observations were reported by Dipalma et al. [39], who described improved oral hygiene maintenance and reduced inflammatory parameters in aligner-treated patients, while also emphasizing the heterogeneity and risk of bias of the available evidence and the importance of patient-specific clinical decision-making.

Regarding inflammatory biomarkers, cytokine levels in GCF showed variable temporal patterns across studies. Proinflammatory cytokines (IL-1β, TNF-α, and MMP-8) were assessed at different timepoints, though inconsistent sampling schedules complicate interpretation [11,29]. This cautious interpretation is further supported by the study of Nemec et al. [40], who reported no significant longitudinal changes in salivary inflammatory markers or overall microbial community composition during orthodontic treatment with either aligners or fixed appliances in patients maintaining adequate oral hygiene.

Periodontal assessment exhibited considerable divergence in approach: diverse indices were utilized (Silness–Löe, Turesky modification, O’Leary) alongside disparate follow-up intervals (1–12 months). Examiner calibration and standardized timing relative to aligner wear were infrequently documented, thereby constraining evidence synthesis.

In vitro studies addressed thermoplastic aligner materials (polyurethanes, PETG, and medical-grade polyesters) without cytotoxic responses in human gingival fibroblasts and oral epithelial cells [29]. However, saliva from patients undergoing orthodontic treatment (both aligners and brackets) increased expression of proinflammatory markers (IL-6, IL-8, MCP-1) in gingival fibroblasts, with no significant difference between treatment modalities [29]. These results were presented within laboratory-based experimental contexts and were not directly linked to clinical outcome measures. One study discussed the potential release of residual compounds after repeated thermal cycles and noted periodic replacement within their protocols [28].

For aligner trimline, a small number of comparative studies outlined plaque accumulation and local gingival response in relation to supragingival and juxtagingival configurations. These findings are based on limited, methodologically diverse evidence and should be interpreted as hypothesis-generating rather than practice-directing [5].

Both clinical and laboratory investigations were frequently characterized by small sample sizes, short observation periods, and non-uniform outcome measures, alongside experimental conditions that do not fully replicate the clinical oral environment, thereby affecting external validity.

The limited evidence specifically addressing aligner marginal edge design underscores the need for targeted investigations and supports the rationale for this scoping review. Notably, included studies predominantly enrolled adolescent and adult patients (aged 12–35 years) with healthy or well-controlled periodontal status; evidence for patients with moderate-to-severe periodontal disease or recent surgical interventions remains scarce. Future research should prioritize longer follow-up periods, standardized periodontal and biomarker assessments, integrated clinical–microbiological designs, and inclusion of periodontally compromised populations to better characterize the clinical relevance of marginal edge design.

## 5. Conclusions

This scoping review mapped clinical and laboratory evidence on periodontal and biological outcomes associated with CA therapy. Twenty-five studies published between 2020 and 2025 were included, employing diverse methodological approaches. Most investigations reported stable or favorable periodontal parameters during aligner therapy in cohorts with adequate oral hygiene, with substantial heterogeneity in assessment protocols and outcome definitions limiting cross-study comparisons.

Evidence on aligner marginal edge design remains scarce, with only three studies addressing trimline configurations and one directly comparing margin types. Current findings preclude definitive conclusions regarding optimal margin design for periodontal health.

Future research should prioritize: standardized periodontal assessment protocols enabling meaningful synthesis; adequately powered comparative studies on marginal edge designs; longitudinal investigations extending beyond active treatment; and inclusion of patients with compromised periodontal status. This review provides a foundation for targeted investigations and supports the development of standardized research protocols in this field.

## Figures and Tables

**Table 1:** Distribution of eligible articles according to the field of interest: gingival/periodontal inflammation, biofilm and inflammatory markers, and aligner margin design.

No.	Article Title	Gingival/ Periodontal Inflammation	Biofilm and Inflammatory Markers	Aligner Margin Design
1	Impact of Fixed Orthodontic Appliance and Clear Aligners on the Periodontal Health: A Prospective Clinical Study [17]	Yes	Yes	-
2	Association between Gingival Phenotype and Periodontal Disease Severity—A Comparative Longitudinal Study Among Patients Undergoing Fixed Orthodontic Therapy and Invisalign Treatment [6]	Yes	-	-
3	Characteristics of Oral Microbiota and Oral Health [18]	Yes	Yes	Partial
4	Comparative Analysis of Alkaline Phosphatase Levels in Gingival Crevicular Fluid: A Three-Time Point Study Comparing Clear Aligners and Fixed Orthodontic Appliances [19]	Yes	Yes	-
5	Clear Aligners—An Efficient Tool in the Combined Ortho-Perio Treatment of Gingival Recessions [20]	Yes	-	-
6	Microbiological Analysis of Plaque and Its Composition in Three Patient Groups under Different Orthodontic Treatments [21]	Yes	Yes	-
7	Comparative Study of Periodontal Health in Patients with Fixed Braces Versus Clear aligners [22]	Yes	Yes	-
8	Dimensional Changes in the Gingival Tissues Induced by Clear Aligners and Fixed Orthodontic Appliances [23]	Yes	-	-
9	Comparison of Cytokine Level Changes in Gingival Crevicular Fluid Between the Aligner and Pendulum Appliance During Early Molar Distalization [9]	Yes	Yes	-
10	Effectiveness of Invisalign^®^ Aligners in the Treatment of Severe Gingival Recession: A Case Report [24]	Yes	-	-
11	Expansion Treatment Using Invisalign^®^: Periodontal Health Status and Maxillary Buccal Bone Changes. A Clinical and Tomographic Evaluation [25]	Yes	-	-
12	Evaluation of IL-8 and IL-6 Levels in Gingival Crevicular Fluid of Individuals Undergoing Clear Aligner Therapy [10]	Yes	Yes	-
13	Edge Level of Aligners and Periodontal Health: A Clinical Perspective Study in Young Patients [5]	Yes	-	Yes
14	Orthodontic Treatment with Fixed Appliances Versus Aligners: An Experimental Study of Periodontal Aspects [26]	Yes	Yes	-
15	Oral Health in Adolescents: Periodontal Inflammatory Biomarkers During Orthodontic Clear Aligner Therapy [11]	Yes	Yes	Mentioned
16	Levels of Inflammatory and Bone Metabolic Markers in the Gingival Crevicular Fluid of Individuals Undergoing Fixed Orthodontic Treatment in Comparison to Those Utilizing Invisalign [27]	Yes	Yes	-
17	MALDI-TOF/MS Profiling of Whole Saliva and Gingival Crevicular Fluid in Patients with the Invisalign System and Fixed Orthodontic Appliances [28]	-	Yes	-
18	Effects of the Saliva of Patients Undergoing Orthodontic Treatment with Invisalign and Brackets on Human Gingival Fibroblasts and Oral Epithelial Cells [29]	-	Yes	-
19	Periodontal Parameters in Adult Patients with Clear Aligners Orthodontics Treatment Versus Three Other Types of Brackets: A Cross-Sectional Study [30]	Yes	-	-
20	Periodontal Outcomes and Digital Data Integration of Orthodontic Treatment with Clear Aligners: A Prospective Pilot Study [31]	Yes	-	-
21	Proton-Nuclear Magnetic Resonance Metabolomics of Gingival Crevicular Fluid During Orthodontic Tooth Movement with Aligners [32]	-	Yes	-
22	Role of Six Cytokines and Bone Metabolism Biomarkers in Gingival Crevicular Fluid in Patients Undergoing Fixed Orthodontic Appliance Treatment in Comparison with Aligners: A Clinical Study [33]	Yes	Yes	-
23	Short-Term Variation in the Subgingival Microbiota in Two Groups of Patients Treated with Clear Aligners and Vestibular Fixed Appliances: A Longitudinal Study [34]	Yes	Yes	-
24	Retrospective Study on Orthodontic Gingival Recession Correction Using Clear Aligners [7]	Yes	-	-
25	Class III Correction and Enhanced Periodontal Health with Aligner Treatment in a 53-Year-Old Patient [8]	Yes	-	-

**Table 2:** Methodological characteristics of the 14 included studies, according to the type of comparison, biomarker monitoring, and clinical evolution.

No.	Article Title	Comparison: Aligners vs. Fixed Appliances	Biomarker/ Biofilm Monitoring	Microbiological/ Immunological Analysis	Clinical Follow-Up/ Evolutionary Phases
1	Impact of Fixed Orthodontic Appliance and Clear Aligners on the Periodontal Health: A Prospective Clinical Study [17]	Yes	Yes	Yes	Yes
2	Association Between Gingival Phenotype and Periodontal Disease Severity—A Comparative Longitudinal Study Among Patients Undergoing Fixed Orthodontic Therapy and Invisalign Treatment [6]	Yes	Partial	Yes	Yes
3	Characteristics of Oral Microbiota and Oral Health in the Patients Treated with Clear Aligners: A Prospective Study [18]	No	Yes	Yes	Yes
4	Comparative Analysis of Alkaline Phosphatase Levels in Gingival Crevicular Fluid: A Three-Time Point Study Comparing Clear Aligners and Fixed Orthodontic Appliances [19]	Yes	Yes	Yes	Yes
5	Microbiological Analysis of Plaque and Its Composition in Three Patient Groups Under Different Orthodontic Treatments [21]	Yes	Yes	Yes	Yes
6	Comparative Study of Periodontal Health in Patients with Fixed Braces Versus Clear Aligners [22]	Yes	Yes	Yes	Yes
7	Dimensional Changes in the Gingival Tissues Induced by Clear Aligners and Fixed Orthodontic Appliances [23]	Yes	Partial	Partial	Yes
8	Comparison of Cytokine Level Changes in Gingival Crevicular Fluid Between the Aligner and Pendulum Appliance During Early Molar Distalization [9]	Yes	Yes	Yes	Yes
9	Expansion Treatment Using Invisalign^®^: Periodontal Health Status and Maxillary Buccal Bone Changes. A Clinical and Tomographic Evaluation [25]	Yes	Yes	Partial	Yes
10	Edge Level of Aligners and Periodontal Health: A Clinical Perspective Study in Young Patients [5]	Yes	Yes	Yes	Yes
11	Orthodontic Treatment with Fixed Appliances Versus Aligners: An Experimental Study of Periodontal Aspects [26]	Yes	Yes	Yes	Yes
12	Oral Health in Adolescents: Periodontal Inflammatory Biomarkers During Orthodontic Clear Aligner Therapy [11]	Yes	Yes	Yes	Yes
13	Levels of Inflammatory and Bone Metabolic Markers in the Gingival Crevicular Fluid of Individuals Undergoing Fixed Orthodontic Treatment in Comparison to Those Utilizing Invisalign [27]	Yes	Yes	Yes	Yes
14	Periodontal Parameters in Adult Patients with Clear Aligners Orthodontics Treatment Versus Three Other Types of Brackets: A Cross-Sectional Study [30]	Yes	Yes	Yes	Yes

**Table 3:** Characteristics of experimental studies on CA, sorted according to type of analysis performed, investigated molecular markers, and testing environment.

No.	Article Title	Cytotoxicity Analysis	Surface/ Material Analysis	Molecular Marker Evaluation (ELISA, PCR, Biochemistry)	Samples/ Testing Environment
1	Comparative Analysis of Alkaline Phosphatase Levels in Gingival Crevicular Fluid: A Three-Time Point Study Comparing Clear Aligners and Fixed Orthodontic appliances [19]	Yes	Yes	Yes	Aligner material, GCF
2	MALDI-TOF/MS Profiling of Whole Saliva and Gingival Crevicular Fluid in Patients with the Invisalign System and Fixed Orthodontic Appliances [28]	Yes	Partial	Yes	Patient saliva, proteomics
3	Effects of the Saliva of Patients Undergoing Orthodontic Treatment with Invisalign and Brackets on Human Gingival Fibroblasts and Oral Epithelial Cells [29]	Yes	-	Yes	Patient saliva, cell cultures
4	Proton-Nuclear Magnetic Resonance Metabolomics of Gingival Crevicular Fluid During Orthodontic Tooth Movement with Aligners [32]	-	Yes	Yes	GCF
5	Role of Six Cytokines and Bone Metabolism Biomarkers in Gingival Crevicular Fluid in Patients Undergoing Fixed Orthodontic Appliance Treatment in Comparison with Aligners: A Clinical Study [33]	-	-	Yes	GCF, aligner material

**Table 4:** Characteristics of interdisciplinary case reports, focusing on pathology complexity, ortho-periodontal approach, and innovative aspects of treatment with CA.

No.	Article Title	Pathology Complexity	Interdisciplinary Approach/Reported Clinical Observations	Innovative/ Regenerative Aspect
1	Class III Correction and Enhanced Periodontal Health with Aligner Treatment in a 53-Year-Old Patient [8]	Class III + severe recessions	Ortho-perio, recession, and tooth mobility management	Stable periodontal outcome, functional correction
2	Effectiveness of Invisalign^®^ Aligners in the Treatment of Severe Gingival Recession: A Case Report [24]	Gingival recessions + dehiscence	Ortho-perio, radiological, and CBCT control	Root migration, bone healing
3	Clear Aligners—An Efficient Tool in the Combined Ortho-Perio Treatment of Gingival Recessions [20]	Multiple recessions	Ortho-perio protocol: combined ortho + surgical treatment	Pre-surgical optimization with aligners

**Table 5:** Studies with an interdisciplinary approach integrating ortho-periodontal clinical evaluation with molecular marker analysis, microbial sequencing, and specialist collaboration.

No.	Article Title	Orthodontic–Periodontal Clinical Evaluation	Molecular Markers (Cytokines, Enzymes)	Microbial Analysis and Genomic Associations	Involved Specialists
1	Characteristics of Oral Microbiota and Oral Health [18]	Yes	No	Yes	Orthodontist, periodontist, microbiologist
2	Oral Health in Adolescents: Periodontal Inflammatory Biomarkers During Orthodontic Clear Aligner Therapy [11]	Yes	aMMP-8 only; cytokine levels not assessed	Yes/genomic (IL-1)	Orthodontist, periodontist, geneticist
3	Orthodontic Treatment with Fixed Appliances Versus Aligners: An Experimental Study of Periodontal Aspects [26]	Yes	Yes	Partial	Orthodontist, periodontist, biochemist
4	Levels of Inflammatory and Bone Metabolic Markers in the Gingival Crevicular Fluid of Individuals Undergoing Fixed Orthodontic Treatment in Comparison to Those Utilizing Invisalign [27]	Yes	Yes	Yes	Orthodontist, periodontist, biochemist
5	Comparative Analysis of Alkaline Phosphatase Levels in Gingival Crevicular Fluid: A Three-Time Point Study Comparing Clear Aligners and Fixed Orthodontic Appliances [19]	Yes	Yes	No	Orthodontist, periodontist, biochemist
6	Comparison of Cytokine Level Changes in Gingival Crevicular Fluid Between the Aligner and Pendulum Appliance During Early Molar Distalization [9]	Yes	Yes	No	Orthodontist, periodontist, biochemist
7	MALDI-TOF/MS Profiling of Whole Saliva and Gingival Crevicular Fluid in Patients with the Invisalign System and Fixed Orthodontic Appliances [28]	Yes	Yes	Yes	Orthodontist, periodontist, biochemist
8	Effects of the Saliva of Patients Undergoing Orthodontic Treatment with Invisalign and Brackets on Human Gingival Fibroblasts and Oral Epithelial Cells [29]	Yes	Yes	No	Orthodontist, periodontist, biochemist
9	Role of Six Cytokines and Bone Metabolism Biomarkers in Gingival Crevicular Fluid in Patients Undergoing Fixed Orthodontic Appliance Treatment in Comparison with Aligners: A Clinical Study [33]	Yes	Yes	No	Orthodontist, periodontist, microbiologist
10	Short-Term Variation in the Subgingival Microbiota in Two Groups of Patients Treated with Clear Aligners and Vestibular Fixed Appliances: A Longitudinal Study [34]	Yes	Yes	Yes	Orthodontist, periodontist, microbiologist

## Data Availability

No new data were created or analyzed in this study.

## Supplementary Materials

The following supporting information can be downloaded at: https://www.mdpi.com/article/10.3390/dj14030130/s1, Supplementary Material S1: PRISMA-ScR Checklist; Supplementary Material S2: Full Search Strategies; Supplementary Material S3: Characteristics of Included Studies; Supplementary Material S4: Articles Excluded with Reasons.

## Abbreviations

The following abbreviations are used in this manuscript:

aMMP-8	Active Matrix Metalloproteinase-8
BOP	Bleeding on Probing
CA	Clear Aligner
CAL	Clinical Attachment Level
ELISA	Enzyme-Linked Immunosorbent Assay
GI	Gingival Index
GCF	Gingival Crevicular Fluid
IL	Interleukin
IL-1β	Interleukin-1 beta
IL-6	Interleukin-6
IL-8	Interleukin-8
MMP-8	Matrix Metalloproteinase-8
PCC	Population–Concept–Context framework
PCR	Polymerase Chain Reaction
PETG	Polyethylene Terephthalate Glycol
PI	Plaque Index
PPD	Probing Pocket Depth
PRISMA-ScR	Preferred Reporting Items for Systematic Reviews and Meta-Analyses Extension for Scoping Reviews
TNF-α	Tumor Necrosis Factor alpha
