# Supplementary material for: Clear Aligner Therapy and Marginal Edge Design: Clinical and Laboratory Evidence on Periodontal and Biological Outcomes—A Scoping Review
Source: Dent J (Basel). 2026 Feb 24;14(3):130. doi: 10.3390/dj14030130 (PMC13025924; doi:10.3390/dj14030130)
Supplement: Supplementary file 1 [file dentistry-14-00130-s001.zip › Supplementary Material S1 PRISMA-ScR checklist DJ.pdf]

Preferred Reporting Items for Systematic reviews and Meta-Analyses extension for Scoping Reviews (PRISMA-ScR) Checklist

SECTION	ITEM	PRISMA-ScR CHECKLIST ITEM	REPORTED ON PAGE #
**TITLE**			
Title	1	Identify the report as a scoping review.	Title page - "A Scoping Review"
**ABSTRACT**			
Structured summary	2	Provide a structured summary that includes (as applicable): background, objectives, eligibility criteria, sources of evidence, charting methods, results, and conclusions that relate to the review questions and objectives.	Abstract - structured into Background, Objectives, Methods, Results, and Conclusions, in accordance with PRISMA-ScR requirements
**INTRODUCTION**			
Rationale	3	Describe the rationale for the review in the context of what is already known. Explain why the review questions/objectives lend themselves to a scoping review approach.	Introduction - paragraphs 2-6 (describes rationale and need for scoping review)
Objectives	4	Provide an explicit statement of the questions and objectives being addressed with reference to their key elements (e.g., population or participants, concepts, and	Introduction - final paragraph (Population-Concept-Context framework and research question)

SECTION	ITEM	PRISMA-ScR CHECKLIST ITEM	REPORTED ON PAGE #
		context) or other relevant key elements used to conceptualize the review questions and/or objectives.	
**METHODS**			
Protocol and registration	5	Indicate whether a review protocol exists; state if and where it can be accessed (e.g., a Web address); and if available, provide registration information, including the registration number.	Methodology - paragraph stating that the review followed the PRISMA-ScR guidelines; no protocol registered; PROSPERO not applicable
Eligibility criteria	6	Specify characteristics of the sources of evidence used as eligibility criteria (e.g., years considered, language, and publication status), and provide a rationale.	2.3 Study integration and selection - inclusion and exclusion criteria described (years 2015-2025; English/Romanian; clinical and lab studies)
Information sources*	7	Describe all information sources in the search (e.g., databases with dates of coverage and contact with authors to identify additional sources), as well as the date the most recent search was executed.	2.2 Search strategy - PubMed (MEDLINE), Scopus, and Web of Science databases used; last search 24 Oct 2025

SECTION	ITEM	PRISMA-ScR CHECKLIST ITEM	REPORTED ON PAGE #
Search	8	Present the full electronic search strategy for at least 1 database, including any limits used, such that it could be repeated.	2.2 Search strategy - MeSH terms and free-text keywords, filters, time frame; full strategies in Supplement S2
Selection of sources of evidence	9	State the process for selecting sources of evidence (i.e., screening and eligibility) included in the scoping review.	2.3 Study integration and selection - two-stage screening (title/abstract, then full-text); records managed in Zotero; collaborative screening by the research team; no automation tools.
Data charting process	10	Describe the methods of charting data from the included sources of evidence (e.g., calibrated forms or forms that have been tested by the team before their use, and whether data charting was done independently or in duplicate) and any processes for obtaining and confirming data from investigators.	2.3 Study integration and selection - data charted and verified collaboratively by the research team (no automation tools).
Data items	11	List and define all variables for which data were sought and any assumptions and simplifications made.	2.1 Study type and aim - periodontal indices, inflammatory markers, microbiological characteristics, aligner margin design variables
Critical appraisal of individual sources of evidence	12	If done, provide a rationale for conducting a critical appraisal of included sources of evidence; describe the methods	Not conducted - critical appraisal optional for scoping reviews

SECTION	ITEM	PRISMA-ScR CHECKLIST ITEM	REPORTED ON PAGE #
		used and how this information was used in any data synthesis (if appropriate).	
Synthesis of results	13	Describe the methods of handling and summarizing the data that were charted.	Results section - opening paragraph ("Data were synthesized descriptively and thematically…"); no meta-analysis performed
**RESULTS**			
Selection of sources of evidence	14	Give numbers of sources of evidence screened, assessed for eligibility, and included in the review, with reasons for exclusions at each stage, ideally using a flow diagram.	Results 3.1 - Study selection; Figure 1 (Flow diagram of the search, deduplication, and study selection process); Supplement S2
Characteristics of sources of evidence	15	For each source of evidence, present characteristics for which data were charted and provide the citations.	Results 3.2 - Study characteristics summarized in Tables 1-5
Critical appraisal within sources of evidence	16	If done, present data on critical appraisal of included sources of evidence (see item 12).	Not applicable - no critical appraisal performed (Scoping Review)
Results of individual sources of evidence	17	For each included source of evidence, present the relevant data that were charted that relate to the review questions and objectives.	Results 3.1-3.3 - Individual study results grouped by themes: gingival/periodontal inflammation, biofilm & markers, edge design
Synthesis of results	18	Summarize and/or present the charting results as they relate	Results 3.3 - Summary describing patterns and thematic synthesis

SECTION	ITEM	PRISMA-ScR CHECKLIST ITEM	REPORTED ON PAGE #
		to the review questions and objectives.	
**DISCUSSION**			
Summary of evidence	19	Summarize the main results (including an overview of concepts, themes, and types of evidence available), link to the review questions and objectives, and consider the relevance to key groups.	Discussion - contextual discussion of mapped evidence of overall findings in context of existing evidence
Limitations	20	Discuss the limitations of the scoping review process.	Discussion - limitations of included evidence and of review processes
Conclusions	21	Provide a general interpretation of the results with respect to the review questions and objectives, as well as potential implications and/or next steps.	Conclusions section - interpretation, implications for future research and evidence development and future research directions
**FUNDING**			
Funding	22	Describe sources of funding for the included sources of evidence, as well as sources of funding for the scoping review. Describe the role of the funders of the scoping review.	This work was supported by the project *FOCUS: Training and Guidance for UMFST Researchers in Health*, contract no. 100455/29.08.2025, project code SMIS 350717. The project is co-funded by the European Union under the Health Programme of the Ministry of Investments and European Projects and implemented through the Managing Authority for the Health Programme, PS/688/PS_P3/OP4/ESO4.7/PS_P3_ESO4.7_A6.

JBI = Joanna Briggs Institute; PRISMA-ScR = Preferred Reporting Items for Systematic reviews and Meta-Analyses extension for Scoping Reviews.

* Where *sources of evidence* (see second footnote) are compiled from, such as bibliographic databases, social media platforms, and Web sites.
† A more inclusive/heterogeneous term used to account for the different types of evidence or data sources (e.g., quantitative and/or qualitative research, expert opinion, and policy documents) that may be eligible in a scoping review as opposed to only studies. This is not to be confused with *information sources* (see first footnote).
‡ The frameworks by Arksey and O'Malley (6) and Levac and colleagues (7) and the JBI guidance (4, 5) refer to the process of data extraction in a scoping review as data charting.
§ The process of systematically examining research evidence to assess its validity, results, and relevance before using it to inform a decision. This term is used for items 12 and 19 instead of "risk of bias" (which is more applicable to systematic reviews of interventions) to include and acknowledge the various sources of evidence that may be used in a scoping review (e.g., quantitative and/or qualitative research, expert opinion, and policy document).

From: Tricco AC, Lillie E, Zarin W, O'Brien KK, Colquhoun H, Levac D, et al. PRISMA Extension for Scoping Reviews (PRISMAScR): Checklist and Explanation. Ann Intern Med. 2018;169:467–473. doi: 10.7326/M18-0850.
