# Supplementary material for: Clear Aligner Therapy and Marginal Edge Design: Clinical and Laboratory Evidence on Periodontal and Biological Outcomes—A Scoping Review
Source: Dent J (Basel). 2026 Feb 24;14(3):130. doi: 10.3390/dj14030130 (PMC13025924; doi:10.3390/dj14030130)
Supplement: Supplementary file 1 [file dentistry-14-00130-s001.zip › Supplementary Material S2 Full search strategies DJ.pdf]

Supplementary Material S2: Full Electronic Search Strategies for PubMed, Scopus, and Web of Science

This supplementary file provides the complete electronic search strategies used in the scoping review "Clear Aligner Therapy and Marginal Edge Design: Clinical and Laboratory Evidence on Periodontal and Biological Outcomes—A Scoping Review." The searches were conducted according to the PRISMA-ScR recommendations and followed the Population-Concept-Context (PCC) framework.

General information

- Search period: January 2015 - October 24, 2025
- Languages: English and Romanian. Romanian-language records were included to minimize language bias and were screened and reported in English where relevant.
- Population: Adolescent and adult orthodontic patients (≥12 years old)
- Concept: Influence of clear aligner therapy and marginal edge design on periodontal parameters, inflammatory biomarkers, and biofilm
- Context: Clinical and laboratory studies in dentistry, periodontology, and orthodontics
- Filters applied: Humans, Articles, Full Text available, English or Romanian language. Full texts were retrieved where available, no exclusion was made solely based on access status.

Database search strategies

Database	Search string (Boolean operators and keywords)	Filters / Limits	Date last searched
PubMed (MEDLINE)	("orthodontic appliances, removable"[MeSH] OR "clear aligner" OR "Invisalign" OR "orthodontic aligner") AND ("periodontal diseases"[MeSH] OR "gingival inflammation" OR "biofilm" OR "microbiome" OR "IL-1β" OR "TNF-α" OR "MMP-8" OR "marginal design" OR "trimline" OR "scalloped edge")	Humans; Articles; English or Romanian; 2015-2025	24 Oct 2025
Scopus	TITLE-ABS-KEY ("clear aligner" OR "Invisalign" OR "orthodontic appliance") AND TITLE-ABS-KEY ("periodontal disease" OR "gingival index" OR "biofilm" OR "microbiome" OR "cytokines" OR "IL-1β" OR "MMP-8" OR "trimline" OR "edge design" OR "scalloped")	Subject area: Dentistry; English; 2015-2025	24 Oct 2025
Web of Science (Core Collection)	("orthodontic appliance" OR "clear aligner" OR "Invisalign") AND ("periodontal disease" OR "gingival index" OR "biofilm" OR "microbiome" OR "cytokine" OR "MMP-8" OR "IL-1β" OR "trimline" OR "scalloped edge")	Subject area: Dentistry, Oral Surgery & Medicine; English; 2015-2025	24 Oct 2025

Notes

• The search strings were adapted to the syntax of each database.

• Excluded topics: sequence alignment, ophthalmology, orthopedics, molecular biology unrelated to dentistry.

• Additional manual searches were performed in reference lists of key articles.

Example reference managers used

Zotero (for deduplication and reference management) and Anara.ai (for literature organization and comprehension).
