# Supplementary material for: Clear Aligner Therapy and Marginal Edge Design: Clinical and Laboratory Evidence on Periodontal and Biological Outcomes—A Scoping Review
Source: Dent J (Basel). 2026 Feb 24;14(3):130. doi: 10.3390/dj14030130 (PMC13025924; doi:10.3390/dj14030130)
Supplement: Supplementary file 1 [file dentistry-14-00130-s001.zip › Supplementary Material S3 Characteristics of the 25 Studies Included in the Scoping Review DJ.pdf]

Supplementary Material S3: Characteristics of the 25 Studies Included in the Scoping Review

This supplementary file summarizes the main characteristics of the 25 studies included in the scoping review "Clear Aligner Therapy and Marginal Edge Design: Clinical and Laboratory Evidence on Periodontal and Biological Outcomes—A Scoping Review." Studies are grouped thematically across periodontal/gingival inflammation, biofilm and inflammatory markers, and aligner marginal design. Key fields include citation, study type, focus, assessed parameters, whether aligner edge design was explicitly considered, and a concise key finding.

No.	Citation (First author, Year)	Study Type	Focus / Objective	Parameters Assessed	Edge Design Mentioned	Key Outcomes Assessed / Evidence Mapped
1	Pango Madariaga et al., 2020 (Dent J) [Ref 16]	Prospective clinical	Compare periodontal health: clear aligners vs. fixed appliances	PI, GI, BOP, PPD, CAL	No	Plaque and gingival related periodontal parameters were assessed during clear aligner and fixed appliance therapy.
2	Alasiri et al., 2024 (Healthcare) [Ref 6]	Longitudinal clinical	Gingival phenotype vs periodontal severity in fixed vs Invisalign	Periodontal indices (PI, GI, BOP, PPD, CAL)	No	Periodontal indices were assessed in relation to gingival phenotype and periodontal severity during clear aligner and fixed appliance therapy.
3	Rouzi et al., 2023 (Clin Oral Investig) [Ref 17]	Prospective clinical	Oral microbiota and oral health in aligner patients	Microbiome composition; clinical oral health indicators	Yes	Subgingival microbiota composition and oral health indicators were examined during clear aligner therapy.
4	Pisarla et al., 2025 (J Pharm Bioallied Sci) [Ref 18]	Clinical biochemical (3 time points)	GCF alkaline phosphatase in aligners vs fixed	GCF ALP; periodontal indices	No	Gingival crevicular fluid alkaline phosphatase levels and periodontal indices were assessed at multiple time points during aligner and fixed appliance therapy.
5	Leibovich et al., 2024 (Semin Orthod) [Ref 19]	Clinical (ortho-perio treatment)	Clear aligners in combined treatment of gingival recessions	Recession measures; clinical periodontal status	No	Gingival recession parameters and clinical periodontal status were evaluated during interdisciplinary orthodontic–periodontal treatment involving clear aligners.
6	Cenzato et al., 2024 (Dent J) [Ref 20]	Clinical microbiology	Plaque microbiology under different orthodontic treatments	Plaque composition; microbial load; periodontal indices	No	Plaque microbiological composition and periodontal indices were examined under different orthodontic treatment modalities.
7	Shashikumar et al., 2024 (J Pharm Bioallied Sci) [Ref 21]	Comparative clinical	Periodontal health in fixed braces vs clear aligners	PI, GI, BOP, PPD, CAL; biofilm metrics	No	Periodontal parameters and biofilm-related metrics were assessed during clear aligner and fixed appliance therapy.

8	Romito et al., 2024 (J Dent) [Ref 22]	Comparative clinical	Dimensional changes of gingival tissues with aligners vs fixed	Gingival dimensions; periodontal soft tissue metrics	No	Dimensional changes of gingival tissues and periodontal soft tissue metrics were assessed during aligner and fixed appliance treatment.
9	Chen et al., 2021 (J Orofac Orthop) [Ref 9]	Prospective observational	Cytokine changes in GCF: aligner vs pendulum during distalization	ILs in GCF; periodontal indices	No	Cytokine levels in gingival crevicular fluid were measured at multiple time points during aligner therapy.
10	Figueiredo et al., 2021 (Korean J Orthod) [Ref 23]	Case report	Effectiveness of Invisalign® in severe gingival recession	Recession depth/width; imaging (CBCT)	No	Gingival recession dimensions and soft tissue parameters were documented during clear aligner therapy.
11	Barreda et al., 2020 (Acta Odontol Latinoam) [Ref 24]	Clinical & tomographic	Expansion with Invisalign®: periodontal status and buccal bone	Clinical periodontal indices; CBCT bone changes	No	Clinical periodontal indices and buccal bone changes were assessed during orthodontic expansion with clear aligners.
12	Altındal et al., 2025 (Angle Orthod) [Ref 10]	Clinical biomarkers	IL-8 and IL-6 levels during aligner therapy	IL-6, IL-8 in GCF; clinical indices	No	Interleukin-6 and interleukin-8 levels in gingival crevicular fluid were measured at different stages of aligner therapy.
13	Favero et al., 2023 (Dent Press J Orthod) [Ref 5]	Clinical perspective study	Edge level of aligners and periodontal health	Gingival/periodontal assessment	Yes	Gingival and periodontal parameters were examined in relation to different aligner marginal edge configurations.
14	Giannini et al., 2025 (Dent J) [Ref 25]	Experimental clinical	Fixed vs aligners: periodontal aspects	Clinical periodontal parameters; biomarkers	No	Clinical periodontal parameters and biomarkers were assessed during clear aligner and fixed appliance therapy.
15	Kredig et al., 2025 (Clin Oral Investig) [Ref 11]	Adolescent clinical biomarkers	Inflammatory biomarkers during aligner therapy in adolescents	Periodontal biomarkers; possible genomic markers	Yes	Periodontal inflammatory biomarkers were evaluated during clear aligner therapy in adolescent patients.
16	Alnazeh et al., 2023 (Medicina) [Ref 26]	Comparative clinical	Inflammatory and bone metabolic markers: fixed vs Invisalign	IL-1β, TNF-α, MMP-8, bone markers in GCF	No	Proinflammatory cytokines and bone metabolic markers in gingival crevicular fluid were assessed during clear aligner and fixed appliance therapy.
17	Bober et al., 2023 (IJERPH) [Ref 27]	Proteomics (MALDI-TOF/MS)	Saliva and GCF profiling: Invisalign vs fixed	Protein markers in saliva and GCF	No	Proteomic profiles in saliva and gingival crevicular fluid were analyzed during clear aligner and fixed appliance therapy.
18	Nemec et al., 2023 (J Clin Med) [Ref 28]	In vitro / cell culture	Effects of patients' saliva (aligners vs brackets) on cells	Gingival fibroblasts & oral epithelial cell responses	No	Cellular responses of gingival fibroblasts and oral epithelial cells exposed to saliva from orthodontic patients were evaluated in vitro.
19	Mulla Issa et al., 2020 (J Orthod Sci) [Ref 29]	Cross-sectional clinical	Periodontal parameters:	PI, GI, BOP, PPD, CAL	Yes	Periodontal parameters were assessed across

			aligners vs three bracket types			clear aligners and different bracket systems.
20	Ravera et al., 2024 (Appl Sci) [Ref 30]	Prospective pilot	Periodontal outcomes and digital data integration with aligners	Clinical periodontal indices	No	Periodontal indices were monitored longitudinally during clear aligner therapy.
21	Domini et al., 2025 (Orthod Craniofac Res) [Ref 31]	Metabolomics (NMR)	GCF metabolomics during tooth movement with aligners	Metabolites in GCF	No	Metabolomic profiles of gingival crevicular fluid were analyzed during tooth movement with clear aligners.
22	Kamran et al., 2023 (Angle Orthod) [Ref 32]	Clinical biomarkers	Six cytokines and bone metabolism markers: fixed vs aligners	Cytokines and bone markers in GCF	No	Cytokine and bone metabolism markers in gingival crevicular fluid were assessed during clear aligner and fixed appliance therapy.
23	Lombardo et al., 2021 (Orthod Craniofac Res) [Ref 33]	Longitudinal microbiology	Short-term variation in subgingival microbiota: aligners vs fixed	Subgingival microbiota; plaque samples	No	Short-term variations in subgingival microbiota were examined during clear aligner and fixed appliance therapy.
24	Bucur et al., 2024 (Medicina) [Ref 7]	Retrospective clinical	Orthodontic gingival recession correction with clear aligners	Gingival recession measures; periodontal indices	Yes	Changes in gingival recession parameters were documented during clear aligner therapy, stratified by baseline severity.
25	Almagrami et al., 2024 (Angle Orthod) [Ref 8]	Case report	Class III correction and periodontal health with aligners (adult)	Clinical periodontal status; functional outcomes	No	Clinical periodontal status and functional outcomes were documented during clear aligner therapy in an adult patient.

The references cited in this table correspond to those listed in the main manuscript.
