# Supplementary material for: Clear Aligner Therapy and Marginal Edge Design: Clinical and Laboratory Evidence on Periodontal and Biological Outcomes—A Scoping Review
Source: Dent J (Basel). 2026 Feb 24;14(3):130. doi: 10.3390/dj14030130 (PMC13025924; doi:10.3390/dj14030130)
Supplement: Supplementary file 1 [file dentistry-14-00130-s001.zip › Supplementary Material S4 Full-text Articles Excluded with Reasons DJ.pdf]

Supplementary Material S4: Full-text Articles Excluded with Reasons (PRISMA-ScR Item 16b)

Corresponds to PRISMA-ScR Checklist Item 16b (Full-text articles excluded with reasons).

The table below reports all articles retrieved in full text but excluded after detailed eligibility assessment. Each article is assigned one primary reason for exclusion, according to the predefined inclusion/exclusion criteria of the review. Laboratory-based (in vitro or ex vivo) studies were excluded when they focused exclusively on material properties or microbial growth without assessment of periodontal, inflammatory, or host biological responses relevant to the review objectives.

Reasons follow PRISMA-ScR standard categories (one primary reason per record).

No.	Article title	Study type	Reason for exclusion
1	Treatment of a Gingival Recession With In-Office Orthodontic Aligners and Gingival Graft.	Case report	Single case; no biomarker or microbiological data.
2	The Optimal Orthodontic Displacement of Clear Aligner for Mild, Moderate and Severe Periodontal Conditions: An in Vitro Study in a Periodontally Compromised Individual Using the Finite Element Model.	Computational (finite element) study	No clinical or biomarker data; purely mechanical analysis.
3	The Incidence and Severity of Open Gingival Embrasures in Adults Treated with Clear Aligners and Fixed Appliances: A Retrospective Cohort Study.	Retrospective cohort study	No periodontal indices or biomarkers measured.
4	The Impact of Adding Chitosan Nanoparticles on Biofilm Formation, Cytotoxicity, and Certain Physical and Mechanical Aspects of Directly Printed Orthodontic Clear Aligners.	In vitro study	Focused on material-related microbiological and cytotoxicity outcomes without periodontal or inflammatory biomarker assessment.
5	The Displacement of Teeth and Stress Distribution on Periodontal Ligament under Different Upper Incisors Proclination with Clear Aligner in Cases of Extraction: A Finite Element Study.	Computational (finite element) study	In silico study without biological parameters.

No.	Article title	Study type	Reason for exclusion
6	Surgically Facilitated Orthodontics with Clear Aligners for Severe Malocclusion and Gingival Recessions.	Case report	Single case; no biomarker or microbiological data.
7	Predictability of the Gingival Margin of the Upper Incisors with Transparent Aligners: A Pilot Retrospective Study.	Retrospective pilot study	Does not report periodontal or inflammatory parameters.
8	Predictability of Incisal Labiolingual Inclination, Overjet, and Overbite Changes, and the Prevalence of Open Gingival Embrasures in Patients with Mandibular Incisor Extraction Treated with Invisalign: A Retrospective Cohort Study.	Retrospective cohort	No clinical periodontal or inflammatory parameters.
9	Periodontal disease treated with clear aligner. (Chinese)	Clinical article	Full-text available only in Chinese; excluded due to language restrictions.
10	Orthognathic Surgery and Aligners. A Comparative Assessment of Periodontal Health and Quality of Life in Postsurgical Orthodontic Treatment with Aligners versus Traditional Fixed Appliances: A Randomized Controlled Trial.	Randomized controlled trial	Focus on post-orthognathic surgery; not generalizable to standard clear aligner therapy.
11	Microbiome and Metabolome Associated with White Spot Lesions in Patients Treated with Clear Aligners.	Cross-sectional clinical study	No periodontal indices or cytokine evaluation; focuses on WSLs.
12	Isolation of Clinical Microbial Isolates during Orthodontic Aligner Therapy and Their Ability to Form Biofilm.	Cross-sectional microbiological study	Microbiological focus only; no clinical periodontal indices or inflammatory biomarkers assessed.
13	IPR Treatment and Attachments Design in Clear Aligner Therapy and Risk of Open Gingival Embrasures in Adults.	Retrospective clinical analysis	Focused on open gingival embrasures; no periodontal or inflammatory biomarker assessment.
14	Invisalign ClinCheck Can Predict Open Gingival Embrasures in Adult Extraction Cases: A Pilot Study.	Retrospective pilot study	Evaluates OGE prediction; lacks periodontal or biomarker analysis.

No.	Article title	Study type	Reason for exclusion
15	Influence of Periodontal Ligament Heights on Sequential and Simultaneous Maxillary Molar Distalization Using Clear Aligners.	Computational (finite element) study	Computational modeling without clinical, periodontal, or biological outcome assessment.
16	In Vitro Evaluation of Biofilm Formation by Streptococcus Mutans and Candida Albicans in Orthodontic Aligners.	In vitro experimental study	Focused on in vitro microbial growth without assessment of periodontal, inflammatory, or host biological responses.
17	Histological Response of the Periodontal Tissue to Mandibular Incisor Movement Using Clear Aligners of Varying Thickness in a Rabbit Model.	Animal model study	Conducted on rabbits; no human periodontal or inflammatory data.
18	Gingival Margins' Modifications during Orthodontic Treatment with Invisalign First®: A Preliminary Study.	Prospective preliminary study	No periodontal indices or biomarker data; pediatric population only.
19	Effects of Various Micro-Osteoperforation Approaches on Canine and Aligner Displacements, and the Stress in the Periodontal Ligament: A Finite Element Analysis.	Computational (finite element) study	No human participants; no periodontal, inflammatory, or host biological outcomes assessed.
20	Effects of Commercially Available Clear Aligner Plastics on Human Gingival Mesenchymal Stem Cells.	In vitro cell culture study	No clinical outcomes or biomarker analysis.
21	Effects of Aligner Activation and Power Arm Length and Material on Canine Displacement and Periodontal Ligament Stress: A Finite Element Analysis.	Computational (finite element) study	No biological or clinical data; mechanical modeling only.
22	Comparison of Microbial Adhesion and Biofilm Formation on Different Orthodontic Aligners.	In vitro experimental study	Focused on microbial adhesion without assessment of periodontal clinical parameters or inflammatory biomarkers.
23	Comparative Characterization of Supragingival Plaque Microbiomes in Malocclusion Adult Female Patients Undergoing Orthodontic Treatment with Removable Aligners or Fixed Appliances: A Descriptive Cross-Sectional Study.	Descriptive cross-sectional study	Only microbiological parameters reported; no clinical periodontal indices or inflammatory biomarkers.

No.	Article title	Study type	Reason for exclusion
24	Comparative Analysis of Stress in the Periodontal Ligament and Center of Rotation in the Tooth after Orthodontic Treatment Depending on Clear Aligner Thickness-Finite Element Analysis Study.	Computational (finite element) study	Computational modeling; no clinical periodontal or biomarker data.
25	Comparative Analysis of Periodontal Pain and Quality of Life in Patients with Fixed Multibracket Appliances and Aligners (Invisalign®): Longitudinal Clinical Study.	Prospective clinical study	Focused on pain and QoL; no inflammatory or periodontal parameters.
26	Comparative Analysis of Periodontal Pain According to the Type of Precision Orthodontic Appliances: Vestibular, Lingual and Aligners. A Prospective Clinical Study.	Prospective clinical study	Focused on pain and QoL; no inflammatory or periodontal parameters.
27	Clinical study of digital clear aligner treatment of 33 adult periodontal disease patients with malocclusion. (Chinese)	Clinical study	Full-text available only in Chinese; excluded due to language restrictions.
28	Clinical Effectiveness of Regenerative Periodontal Surgery and Orthodontic Tooth Movement with Clear Aligners in Stage IV Periodontitis: A Case Series.	Case series	Does not measure inflammatory cytokines or biomarkers.
29	Clear Aligners and Open Gingival Embrasures: Retrospective Study of Epidemiology and Risk Factors.	Retrospective cohort study	Focused on open gingival embrasures; no periodontal or inflammatory biomarker assessment.
30	Biomechanical Effects of Periodontal Status on Molar Sequential Distalization with Clear Aligners: A Finite Element Study.	Computational (finite element) study	No human or biomarker data; purely computational.
31	Biological Safe Gold Nanoparticle-Modified Dental Aligner Prevents the Porphyromonas Gingivalis Biofilm Formation.	In vitro experimental study	Focused on material-related biofilm formation without assessment of periodontal, inflammatory, or host biological responses relevant to the review objectives.
32	Biofilm Formation of Streptococcus Mutans, Streptococcus Sanguinis, Staphylococcus Epidermidis, Staphylococcus Aureus,	In vitro experimental study	Focused on material-related biofilm formation without assessment of periodontal,

No.	Article title	Study type	Reason for exclusion
	Lactobacillus Casei, and Candida Albicans on 5 Thermoform and 3D Printed Orthodontic Clear Aligner and Retainer Materials at 3 Time Points: An in Vitro Study.		inflammatory, or host biological responses relevant to the review objectives.
33	An in Vivo Evaluation of Clear Aligners for Optimal Orthodontic Force and Movement to Determine High-Efficacy and Periodontal-Friendly Aligner Staging.	Animal model study	Conducted on beagles; no human periodontal or inflammatory data.
34	A Novel Machine-Learning-Based Model for Prediction of Open Gingival Embrasures between Mandibular Central Incisors after Clear Aligners Treatment: A Retrospective Cohort Study.	Retrospective cohort study	Computational model predicting OGE; lacks periodontal indices and inflammatory biomarker data.

Clarification: Full-text excluded studies (n = 34) with documented reasons according to PRISMA-ScR 2018.

The numbering in this table does not correspond to citation numbers in the manuscript. Numbers were assigned solely for organizational purposes within this supplementary file, and reflect no specific ordering or ranking of the excluded records. No Romanian-language records met inclusion criteria after full-text screening.
